# Do overweight workers profit by workplace health promotion, more than their normal-weight peers? Evaluation of a worksite intervention

**DOI:** 10.1186/s12995-015-0068-3

**Published:** 2015-08-01

**Authors:** Stefanie Mache, Sarah Jensen, Stefan Linnig, Reimo Jahn, Mirco Steudtner, Elke Ochsmann, Geraldine Preuß

**Affiliations:** Institute for Occupational Medicine and Maritime Medicine (ZfAM), University Medical Center Hamburg-Eppendorf, Seewartenstrasse 10, 20459 Hamburg, Germany; Institute of Occupational Medicine, Charité – Universitätsmedizin Berlin, Free University and Humboldt University, Thielallee 69-73, 14195 Berlin, Germany; Bremen International Graduate School of Social Sciences, Wiener Straße/Ecke Celsiusstraße, 28359 Bremen, Germany; Institute of Technology & Innovation Management, Helmut Schmidt University, Hamburg, Holstenhofweg 85, 22043 Hamburg, Germany; Institute of Health Care Management, University of Applied Sciences Zwickau, Dr.-Friedrichs-Ring 2a, 08056 Zwickau, Germany

**Keywords:** Health behavior changes, Physical activity, Prevention of overweight, Weight gain program, Workplace health promotion

## Abstract

**Background:**

Worksite health promotion programs have been identified as strongly effective in decreasing body weight and increasing awareness and change in health behavior. Aim of this study is to determine the effects of a multi-component intervention in workplace health promotion.

**Methods:**

In a controlled study trail, 1,573 workers of a logistics company had the chance to participate in a one year worksite health promotion program. Main elements of the multi-component intervention were physical activity training in combination with nutrition counseling. Employees completed a questionnaire at baseline and then again after twelve month. Main outcome variables were changes in body weight and health behaviors. Secondary outcomes were subjective health indicators.

**Results:**

Our results showed preliminary improvements in physical activity and eating behavior among normal weight and overweight/obesity weight groups. No significant weight reduction could be found, only a minimal reduction of BMI. The reduction was larger in the overweight group. Workers considered overweight or obese showed significantly greater body weight loss and changes in eating behavior than workers with a normal weight status. Workers with obesity/overweight scored their general health status significantly lower than their colleagues with normal weight status. No significant improvements were found for overall perception of health status between baseline and follow-up in the BMI-groups.

**Conclusion:**

This 12-month intervention-control study suggests that a well-implemented multi-component workplace health promotion program may support substantial change in health behavior (e.g. nutrition and physical activity). It is indicated that overweight employees may especially profit from such worksite health promotion. An investigation of long-term effects of this multi-component intervention is strongly recommended.

## Background

### Obesity, health and economic costs

During the past decades, the prevalence of overweight and obesity has increased to epidemic proportions in developed countries. In Europe, the prevalence of overweight is currently in the range of 50 - 60 % and the prevalence of obesity about 20 % [1, 2].

Obesity is strongly associated with higher rates of coronary heart disease (CHD), stroke, shorter life expectancy and CHD risk factors (e.g., diabetes) [3, 4]. In consequence, overweight and obesity are associated with an increased risk of morbidity and reduced life expectancy [3, 5] and therefore are correlated with increased healthcare and medical costs [6].

In developed countries, about 2-10 % of the overall health care costs are directly attributable to overweight and obesity [2, 7, 8]. In addition, indirect costs associated with sick leaves and working days lost, lower levels of work productivity, individual (psychological) problems and a reduced quality of life are even greater [9, 10].

Therefore, a persistent need for obesity management strategies to address the increasing prevalence of overweight is present these days [11].

The development of overweight and obesity is the result of a complex interaction of behavioral, environmental, social and economic as well as genetic factors [12–14]. The current environment is characterized by a situation whereby food is plentiful and physical activity levels are low [15]. So, overweight and obesity are most often the result of a continuing body weight gain that is triggered by an imbalance between energy expenditure (e.g. low physical activity) and energy intake (e.g. unhealthy dietary behavior) [16].

Increasing physical activity has therefore become an important public health concern. It is important to understand physical activity patterns and health behavior among normal weight but especially among overweight and obese adults in order to develop implement and evaluate successful health interventions.

### Obesity and worksite health promotion

As long-term consequences of overweight and obesity are burdensome for individuals, employers and society, it is necessary to target health behavior such as physical activity and dietary behavior in worksite health promotion interventions [17]. The workplace is considered a central setting to implement programs and strategies both to promote physical activity and to prevent body weight gain and obesity [18, 19]. Moreover, the place of employment represents a relatively controlled environment and a substantial proportion of the adult population can be reached through worksite interventions [20]. Studies evaluating the effect of worksite health promotion interventions targeting physical activity have shown that physical activity levels can be increased [21]. For example, evidence of this effect was found for exercise training and sport interventions [22, 23]. A recently published review shows evidence for limited to moderate positive effects of educational, environmental and multi-component interventions on dietary behavior [24]. For employers, the possibility of increasing productivity may represent a strong incentive for the implementation of worksite programs [25].

### Aim of this study

The aim of the present study was to evaluate the effectiveness of a one-year worksite multi-component intervention related to body weight groups (normal weight vs. overweight) on weight gain, physical activity, perception of health status, eating behavior and health attitude among workers of a logistics company.

We hypothesize that between baseline (t0) and the end of the intervention (follow-up, t2) there will be:

1. Significant differences in the primary outcomes: “body weight”/”body mass index” (BMI) and “perceived health status” and
2. Significant differences in secondary outcomes: ”physical activity during leisure time”, “food consumption”, “behavioral eating attitudes” and “stages of readiness to change dietary behavior” between the normal weight group and the overweight group.

## Methods

### Study-Design

**Fig. 1:**
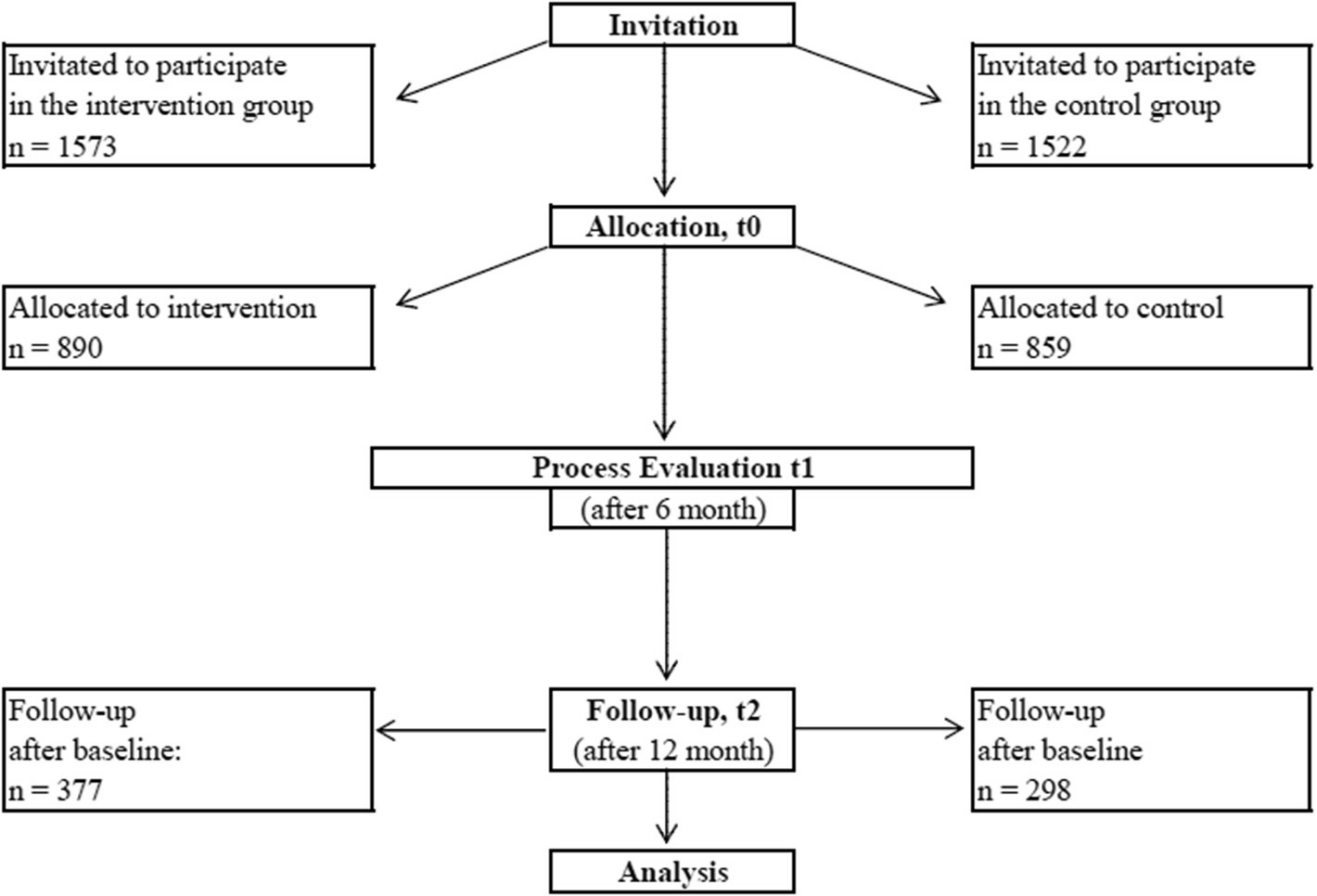
Flow chart

The success of the worksite health intervention was assessed in a controlled study trial. Participants’ ratings were measured at baseline (t0), after six months (t1, process evaluation), and after twelve months (follow-up; t2) (see Fig. 1).

### Outcome evaluation

The data was evaluated at baseline (t0) and at 12 months (t2). Preliminary results of the worksite health intervention related to body weight groups have been assessed on the following types of outcome variables:

1. Participation in training,
2. Satisfaction with training and trainer,
3. Physical activity during leisure time,
4. Changes in weight and body mass index,
5. Nutrition behavioral outcomes (changes in eating behavior, attitudes and stages of readiness to change dietary practices),
6. Perceived health status

### Participants

Throughout the entire year of 2013, 1,573 workers of a German logistics company were invited to participate in multi-component health promotion training (intervention group; ig). In addition, a control group (cg) of 1,522 employees of the same company – comparable to the intervention group in socio-demographic and work-related variables (e.g. gender, age, job description etc.) – has been invited to participate. Groups of workers have been randomized to either the intervention or control group.

In summation, 890 workers of the intervention group (response rate = 58 %) and 859 workers of the control group were involved at baseline (response rate = 56 %). At follow-up (twelve month later), 793 employees of the intervention group and 697 workers of the control group filled-out the survey (response rates of 50 % versus 46 %). Questionnaire data at baseline and follow-up were matched afterwards: It was found, that 377 workers (ig) or 298 (cg), respectively, answered both questionnaires (at baseline and follow-up).

### Worksite Intervention

One of the main aims of this worksite intervention was to encourage health behavior changes in employees, including:

1. fostering motivation towards physical activity,
2. eating healthy food and
3. achieving or maintaining a healthy BMI (<25.0 kg/m^2^).

Secondary goals targeted health behavior and attitude changes.

Participants in the intervention group received a twelve-months health promotion intervention. The training sessions took approx. 30 to 60 minutes every week/fortnight and were held, for example, in a room at the worksite in a group setting of several participants or as an individual coaching. Employees participated during paid working hours; the timetable was set considering the working hours of the staffs (e.g. before working hours, around lunch time and after working hours).

A trainer provided educational information and advice on healthy eating, physical activity, or performed other health promotion activities (e.g. personalized nutrition counseling). Additionally, free fruit and vegetables were provided and physical exercise was included in the sessions at the workplace. Physical activity training focused on general body strength training and included exercises to ease and strengthen muscles at the back, shoulders and arms. Training sessions included practice with flexi bars, barbells, balls and work related objects.

### Measurement/Questionnaires

#### Participation in intervention

Frequency of participation in the intervention was assessed by the following question “How often did you take part in the multi component intervention?”; response options were “frequently” (up to once per week), “irregularly” (two or three times per month) or “never” (no attendance at all).

#### Satisfaction with the intervention/trainer

Participants were asked to rate their overall satisfaction on a five-point Likert-type scale: “All in all, how satisfied are you with the training/trainer?” (1 = very satisfied, 2 = satisfied, 3 = neither…nor, 4 = dissatisfied, 5 = very dissatisfied). High scores correspond to high values on the dimensions.

#### Physical activity during leisure time

In addition, physical activity during leisure time was assessed in the survey. The regularity of physical (sports) activities was specified by categories: regularly (>2 h/week), regularly (≤1-2 h/week), irregularly (≤1 h/week), and no (or almost no) activity.

### Eating behavior/Dietary intake

In order to evaluate individual health behavior, the German version of the *Questionnaire for the Assessment of Health Behaviour*, the FEG (Fragebogen zur Erfassung des Gesundheitsverhaltens), developed by Dlugosch [26] was used. The survey includes several scales and items measuring health risk behavior, health behavior and attitudes towards a healthy diet, smoking, physical activity and the perception of personal well-being. For example, participants are asked to report on frequency of key health behaviors that were emphasized in the program.

In addition, fruit intake was assessed by a short food questionnaire consisting of six questions. Also the intake frequency of fruit, vegetables, sweets (chocolate, cake etc.), fast food, meat products and soft drinks (e.g. cola or lemonade) were assessed by utilizing categorical response options (1 = several times a day, 2 = daily, 3 = several times a week, 4 = seldom, 5 = never) for each category [26]. The survey has been checked for reliability, validity and objectivity by Dlugosch [26].

### Stage of readiness to change eating behavior

The assessment of stage to change eating behavior which is represented by ordered categories of motivational readiness to change (pre-contemplation, contemplation, preparation, action, maintenance) was based on the recommendations by Prochaska and colleagues [27]. For practical reasons, the stages of readiness to change were condensed to four categorical variables of *pre-contemplation*, *contemplation*, *preparation*, and *action/maintenance*. Response options for this question were : “I do not want to change anything/I have no plans to start” (pre-contemplation), “considering a change” (contemplation), “making plans to change” (preparation), “I started doing this” (action, maintenance) [27].

### Eating-related attitudes

Eating-related attitudes and behaviors were measured by the *Questionnaire for the Assessment of Eating-and Weight-Related Attitudes and Behaviors* [28]. Two scales of this questionnaire were included. One of these scales (titled: Attitudes towards healthy eating) consists of 14 items (for example: “I eat lots of vegetables”; “I am very conscious of how much fat is in the food I eat”). Items can be rated on a four-point Likert-type scale (1 = strongly disagree, 2 = disagree, 3 = agree, 4 = strongly agree). Psychometric qualities were acceptable at all measurements (e.g. range of Cronbach’s α = 0.65 to 0.71) [28].

### Employees’ perceived health status

The German version of the *Copenhagen Psychosocial Questionnaire* (COPSOQ) was used to assess employees’ self-rated health status and well-being. Subjective health status was scored on a ten-point rating scale based on the EQ-5D and was transformed to a scale ranging from 0 (“worst health status score”) to 100 points (“best health status score"). Previous investigations successfully proved the quality criteria of the COPSOQ [29]. High scores correspond to high values on the respective dimensions. Thus, in most cases high levels represent a “good” or, in this case, “healthy” status.

### Covariates

At baseline, data on potential effect modifiers and confounders were assessed including age, gender, marital status (assessed in the categories of married; partnership; single; divorced; widowed). Body height and body weight were also measured. Weight status has been evaluated based on three categories of Body Mass Index (BMI): healthy body weight BMI (<25 kg/m^2^); overweight BMI (≥25 kg/m^2^) and obesity BMI (≥30 kg/m^2^). BMI is calculated by dividing body weight in kilograms by the square of the body height in meters and has been measured from self-reported height and weight at baseline (t0) and follow-up (t2).

### Statistical analyses

Descriptive statistics were used to characterize baseline demographics as well as behavioral, cognitive and weight loss outcomes. Normal distribution has been proved by the Shapiro-Wilk test of normality. Chi-square and t-tests revealed significant differences in and associations of socio-demographics, eating behaviors, health attitudes and subjective health status with weight status (i.e., normal weight vs. overweight/obese). In addition, linear mixed effect models were performed with the outcome measures as the dependent variable, group (intervention vs. control group) as independent variable and time of follow-up measurements (t2: follow up at twelve months) as fixed factor, while adjusting for the baseline levels of the outcome measure. A p-level of < 0.05 was considered to indicate statistical significance. All statistical analyses were performed using IBM SPSS (Version 21).

## Results

### Baseline characteristics of the study participants

Table 1 presents the characteristics of the participants divided into bodyweight groups. Most of the participants in the intervention group were in the age groups between 41 and 60 years (73.4 %) (see Table 1). Mean age of the participants was 44 years (SD = 10.26). Gender distribution of the whole sample was approximately equally divided in the groups: 45 % were female, 55 % were male workers. The majority of intervention group (ig) had a normal body weight, with mean BMI being 25.9 (SD = 3.6) and mean BMI of the control group being 25.6 (SD = 3.4). At baseline, 55 % of the participants (ig) or 52 % (cg), respectively, had a normal weight status; 34 % were overweight (cg: 35 %) (BMI > 25 kg/m2); 12 % were obese (cg: 11 %). Chi-square tests show that no significant difference exist between the intervention group (ig) and control group (cg) at baseline with regard to age, gender or other relevant socio-demographic factors.

**Table 1:** Selected demographic characteristics of matched participants

Characteristic	Matched sample participants^a^(Normal weight^b^) (n = 201)	Matched sample participants (Overweight^c^) (n = 169)	Matched sample control group(Normal weight^b^) (n =158 )	Matched sample control group(Overweight^c^) (n = 135)
Age, years, %				
21-30	6.0	8.3	8.2	3.0
31-40	17.4	18.9	12.7	12.6
41-50	44.3	30.8	47.5	45.2
51-60	29.4	39.1	30.4	38.5
>60	1.0	1.2	1.3	0.7
Sex, %				
Male	36.8	43.5	24.1	32.6
Female	58.7	51.8	74.7	63.7
BMI^1^, kg/m^2^				
T0: Mean (SD^1^)	22.6 (1.6)	28.6 (2.5)	22.3 (1.8)	28.6 (2.1)
T2: Mean (SD)	22.7 (1.8)	28.1 (2.9)	22.6 (1.7)	28.7 (2.4)

^1^Abbreviations: BMI - body mass index; SD - standard deviation

^a^Matched sample participants represent the workers identified by code, age, sex, and height with a completed assessment at baseline and follow-up

^b^Normal weight = BMI ≤ 24.9 kg/m^2^

^c^Overweight = BMI ≥ 25.0 kg/m^2^

### Participation in the worksite intervention

Body weight groups did not differ significantly with regard to participation in the intervention program (P = .38). 36.9 % of the overweight group (n = 169) reported that they “frequently” participated in the intervention program (n = 62); 37.5 % participated “irregularly” (n = 63). Only 24.4 % did not participate in the intervention program at all (n = 41). In comparison, 37.9 % of the normal weight group (n = 201) reported that they took part “frequently”, 41.0 % (n = 76) “irregularly”. 21.0 % of these participants reported that they did not take part in the intervention program at all (n = 42).

### Satisfaction with training and trainer

The overweight group showed a mean of 1.89 (SD = 1.23) for satisfaction with the training in total and a mean of 2.15 (SD = 1.73) for satisfaction with the trainer. In comparison, the normal weight group evaluated the training also with a mean of 1.89 (SD = 1.25) and mean of 1.90 (SD = 1.47) for satisfaction with the trainer. No significant difference was found between body weight groups with regard to satisfaction with the intervention (P = 0.79) or trainer (P = 0.57).

### Physical activity during leisure time

As illustrated in Table 2, improvements have been found regarding regularity of physical activity during leisure time between the weight groups.

**Table 2:** Physical activity during leisure time

	Matched sample participants^a^(Normal weight^b^) (n = 201) baseline (%)	Matched sample participants (Overweight^c^) (n = 169) baseline (%)	Matched sample participants(Normal weight^b^) (n =201) follow-up (%)	Matched sample participants (Overweight^c^) (n = 169) follow-up (%)
Physical activity				
no activity	46.2	54.1	39.6	45.2
almost no activity	11.8	15.1	14.2	19.2
irregularly ≤1 h/week	4.1	1.4	17.8	8.2
regularly 1–2 hours per week	26.0	17.8	11.2	10.3
regularly, more than 2 hours per week	11.8	11.6	17.2	17.1

^a^Matched sample participants represent the workers identified by code, age, sex, and height with a completed assessment at baseline and follow-up

^b^Normal weight = BMI ≤ 24.9 kg/m^2^

^c^Overweight = BMI ≥ 25.0 kg/m^2^

Participants in both weight groups reported a more regular physical activity rate at follow-up than at baseline measurement (P = .01).

#### Weight Loss: Changes in body weight and body mass index

Results of measurements at baseline and after twelve-month show that both body weight groups (normal and overweight weight status) in the intervention group did not significantly lose body weight with regard to baseline measurement data. Age- and gender-adjusted BMI changes for the nw-group were −0.21 (CI (95 %) -0.49; −0.15) kg/m^2^ and for the overweight group: −0.32 (CI (95 %) -0.55; −0.12) kg/m^2^ at follow-up. After twelve months, 35 % of the participants (ig) were overweight (cg: 32 %), 12 % were obese (cg: 14 %), 52 % had a normal weight (cg: 53 %).

### Changes in dietary intake and specific eating attitudes

We evaluated changes in eating behaviors between baseline and follow-up. Table 3 shows changes (means and standard deviations at baseline and follow-up measurements) for the targeted dietary intake and eating behaviors among body weight groups. With regard to changes in dietary intake, it was found that participants with normal weight status (BMI < 25) differ from participants with overweight status (BMI > 25). As illustrated in Table 3, participants who are overweight/obese showed greater improvements in consumption of fruit/vegetables and fast food between t0 and t2 than workers who had a normal weight. In general, both groups showed several significant improvements differences in food consumption between baseline and follow-up measurement (P > 0.05).

**Table 3:** Statistical differences between weight status groups (normal weight vs. overweight/obese)

How often do you eat or drink . . . ?	% at Baseline normal weight (n = 198)	% at Baseline overweight/obese (n = 170)	% at Follow-up normal weight (n = 198)	% at Follow-up overweight/obese (n = 170)	*P -*Value^a^
Vegetables					
several time a day	3.1	3.0	8.6	4.7	P(nw^b^) = .05
					P(ow^c^) = .01
daily	28.0	29.8	33.8	44.7	
several times a week	47.7	53.0	48.0	40.6	
less often	13.0	7.1	8.1	8.8	
never	0	0	0	0	
Fruits					
several time a day	8.3	9.5	14.1	14.2	P(nw) = .04
					P(ow) = .01
daily	37.3	35.7	40.4	44.7	
several times a week	29.0	24.4	26.8	28.2	
less often	16.6	22.6	16.7	11.8	
never	1.0	1.8	1.5	0.6	
Sweets					
several time a day	6.7	3.0	6.1	4.7	P(nw) = .00
					P(ow) = .32
daily	24.4	19.6	22.7	18.8	
several times a week	35.8	42.9	41.4	44.1	
less often	24.3	28.0	26.8	28.8	
never	1.0	0.6	1.5	0.6	
Meat					
several time a day	8.3	9.5	7.1	4.1	P(nw) = .00
					P(ow) = .00
daily	31.6	41.7	42.9	46.5	
several times a week	43.0	35.7	36.4	34.7	
less often	7.8	6.0	12.1	12.9	
never	1.0	0.6	1.5	0.6	
Fast Food					
several time a day	0	0	0	0	P(nw) = .00
					P(ow) = .00
daily	0	0.6	0	0	
several times a week	5.7	7.7	7.6	7.1	
less often	76.2	78.0	78.3	84.1	
never	9.3	7.1	12.1	8.8	
Soft drinks (coca-cola, limonade)					
several time a day	3.0	3.5	3.5	0.6	P(nw) = .34
					P(ow) = .53
daily	12.1	10.6	7.1	10.6	
several times a week	13.6	19.4	15.7	20.0	
less often	46.5	43.5	43.4	42.4	
never	22.2	22.4	27.3	24.7	

Notes: χ2 test used for unadjusted comparisons between baseline and one-year follow-up,

^a^P < .05 is significant

^b^nw – normal weight

^c^ow – overweight/obese

In addition changes in eating attitudes have been analyzed (see Tables 4 and 5). At baseline, attitudes to healthy eating were similar in both body weight groups (intervention and control group). At follow-up, few significant differences and improvements in health attitude between the body weight groups (baseline (t0) and follow-up (t2)) were found (see Tables 4 and 5).

**Table 4:** Changes in eating attitudes between participants with normal weight vs. overweight

What do you want to change …	at Baseline (M/SD)^1^ normal weight (n = 198)	at Baseline (M/SD) overweight/obese (n = 170)	at Follow-up (M/SD) normal weight (n = 198)	at Follow-up (M/SD) overweight/obese (n = 170)	*P* Value^a^
1…eating less.	1.54 (1.70)	2.12 (2.11)	1.91 (1.57)	2.16 (2.20)	P(nw^b^) = .01
					P(ow^c^) = .83
2… eating more regularly.	1.69 (2.64)	2.62 (2.21)	1.95 (2.50)	2.55 (2.36)	P(nw) = .17
					P(ow) = .98
3… eating less snacks between the meals.	1.0(2.14)	2.46 (2.24)	1.38 (2.20)	2.39 (2.30)	P(nw) = .02
					P(ow) = .86
4…taking more time for my meals.	1.70 (2.7)	2.41(2.27)	2.12 (2.61)	2.76 (2.35)	P(nw) = .03
					P(ow) = .04
5…eating healthier.	1.60 (2.61)	2.81 (2.23)	1.91 (2.49)	2.65 (2.33)	P(nw) = .11
					P(ow) = .60
6…loose weight.	1.10 (2.02)	2.99 (2.38)	1.11 (2.12)	2.79 (2.45)	P(nw) = .06
					P(ow) = .50

^1^Abbreviations: M- mean; SD- standard deviation

^a^P < .05 is significant

^b^nw – normal weight status

^c^ow – overweight/obese status

**Table 5:** Selected attitudes related to eating behavior: differences between weight groups at baseline and follow-up

Which statements are correct for you?	at Baseline (M/SD)^1^ normal weight (n = 198)	at Baseline (M/SD) overweight/obese (n = 170)	at Follow-up (M/SD) normal weight (n = 198)	at Follow-up (M/SD) overweight/obese (n = 170)	P Value^a^
1. With nutrition I avoid everything that could affect/damage my health.	2.57(0.74)	2.51(0.76)	2.67(0.73)	2.46(0.86)	P(nw^b^) = .11
					P(ow^c^) = .37
2. I always eat healthy and are on a balanced diet.	2.73(0.72)	2.52(0.72)	2.70(0.73)	2.56(0.72)	P(nw) = .64
					P(ow) = .55
3. I place great value on healthy food.	2.68(0.78)	2.61(0.76)	2.73(0.84)	2.65(0.76)	P(nw) = .47
					P(ow) = .46
4. I can say for myself that I eat healthy.	2.75(0.69)	2.58(0.73)	2.78(0.77)	2.57(0.89)	P(nw) = .56
					P(ow) = .85
5. I barely eat unhealthy things.	2.59(0.78)	2.37(0.78)	2.49(0.87)	2.46(0.83)	P(nw) = .17
					P(ow) = .28
6. I eat lots of vegetables.	2.90(0.78)	2.76(0.85)	2.91(0.77)	2.81(0.84)	P(nw) = .80
					P(ow) = .49
7. I eat lots of fruits.	2.81(0.93)	2.79(1.01)	2.79(0.93)	2.71(0.92)	P(nw) = .67
					P(ow) = .26
8. A low fat diet is important to me.	2.65(1.06)	2.61(0.89)	2.58(1.00)	2.58(0.93)	P(nw) = .34
					P(ow) = .77
9. I pay attention for my daily diet to have lots of vitamins and minerals.	2.70(0.78)	2.60(0.79)	2.67(0.85)	2.70(0.70)	P(nw) = .53
					P(ow) = .09

^1^Abbreviations: M - mean; SD - standard deviation

^a^P < .05 is significant

^b^nw – normal weight status

^c^ow – overweight/obese status

### Stage of readiness to change for healthy eating among body weight groups

Significant differences between participants with normal weight (nw; n = 201) and overweight (ow; n = 170) were found regarding stage of readiness to change for health eating.

As illustrated in Table 6, for participants with normal weight, 21 % of the workers were in the preparation stage and 7 % were in the action or maintenance stage at baseline. At follow-up, the percentage of participants in the preparation stage increased to 32 % and the percentage in the action or maintenance stage increased to 14 % (P = 0.001).

**Table 6:** Percentage of participants (intervention group) in stages of readiness to change model from baseline to follow-up by eating behavior

	Stage of change				
	Pre-contemplation	Con- templation	Preparation	Action/maintenance	P Value
*Intervention group*					
Normal weight (BMI < 25) (n = 198)					
Baseline (%)	41.3	25.9	20.9	7.0	P = .000
Follow-up (%)	38.3	13.4	31.8	14.4	
Overweight (BMI > 25) (n = 170)					
Baseline (%)	17.1	34.1	35.3	8.2	P = .000
Follow-up (%)	15.9	17.6	52.9	12.4	
*Controll group*					
Baseline (%)	35.6	29.9	24.2	6.0	P = .003
Follow-up (%)	32.2	15.8	41.3	7.4	

Notes: χ2 test used for unadjusted comparisons between baseline and one-year follow up. P < .05 is significant. Amount

of missing values are not described: thus percentages across columns do not add up to 100 percent

For workers in the overweight group, 35 % were in the preparation stage and 8 % were in the action or maintenance stage at baseline. At follow-up, the percentage of participants (ow) in the preparation stage increased to 53 %; the percentage in the action or maintenance stage increased to 12 % (P = .001).

### Subjective health status

At baseline, participants rated their subjective health status with a mean of 6.36 (SD = 1.90). Correlation analysis showed a significant negative correlation between BMI and subjective health status (r = −.18, P = 0.01). Workers with overweight and obesity rated their perceived health status worse than did workers with a normal weight status. In line with that, stratification showed a significant difference in health status between employees with normal weight status (t0: M = 6.5; SD = 1.9; t2: M = 6.6; SD = 1.8) and employees with overweight status (t0: M = 6.0; SD = 1.8; t2: M = 6.1, SD = 1.9) in the intervention group (P = 0.01).

For the total study population, we found no significant improvements in perceived health status between baseline and follow-up, neither in the intervention nor in the control group (intervention group: M = 6.30, SD = 1.9 vs. M = 6.4, SD = 1.9; P = 0.53; control group: M = 6.17, SD = 1.77 vs. M = 6.21, SD = 1.78, P = 0.69). No interaction between intervention and baseline health status was noted (P > .05).

## Discussion

This study focused on differences among body weight groups regarding changes of body weight, nutrition behavior and health perceptions after a one-year worksite health promotion program that includes physical exercise training in combination with educational nutrition counseling during working time. In total, we found preliminary effects twelve months after the beginning of the intervention, as discussed in the following part.

### Weight gain among BMI-groups

In the beginning, over 40 % of the 890 workers were overweight or obese. This situation indicated a strong need for workplace health promotion. We assumed a significant weight loss after a twelve-month worksite health promotion in both body weight groups. However, body weight did not decrease significantly in both weight groups. Only few participants reported a sizeable weight loss in the overweight group. In comparison to studies targeting similar participants or using comparable intervention methods our findings show comparable results [30, 31]. However, other workplace health promotion studies aiming at weight loss in different body mass index groups illustrated weight losses from 0.5 to 4.0 kg [32–34].

In sum, our results indicate that a worksite health program may lead to weight loss and support former recommendations of combining diverse initiatives for successful weight loss [17]. However, the long-term effects of this combined intervention remain to be investigated. As reported by van Berkel and colleagues, long term effectiveness in weight loss should be central for health interventions, since reaching and maintaining a changed behavioral pattern is challenging, especially for weight loss [35, 36]. To increase long-term success in weight loss, workplace health promotion should be integrated as an inherent part in the company.

### Physical activity among BMI-groups

Our results demonstrate an explicit improvement in physical activity among the BMI-groups as a result of the intervention. Moreover, results indicated that especially overweight employees took advantage of the training: a more regular physical activity is one of their health benefits.

Our findings are comparable to other studies which have also shown an increase in physical activity especially in overweight employees [37–40]. The current public health recommendations advocate regular physical activity as a key component in the prevention and treatment of excess body weight [41]. However, the promotion and modification of physical activity requires an understanding of current activity patterns among normal-weight and overweight adults. Our study provides insight into important issues of how employees spent time in physical activity that can be used to develop programs towards those activities, intensity levels and periods of the week that are especially concerning overweight and/or obese individuals. In addition, our results indicate that the time spent in sport activity differs across BMI categories, suggesting that these should continue to be targets for interventions. However, from a clinical point of view, extreme sport activity should not be prescribed to individuals who are obese and/or not used to exercise [42]. A good approach seems to be to integrate activity into the daily routine, for example by substituting sedentary/sitting and light activities for moderate activities. Moreover, training should be adapted to meet individual needs and preconditions/requirements.

### Changes in nutrition behavior among BMI groups

Furthermore, we hypothesized significant changes in eating behavior after twelve months of worksite health intervention. As a result, we found significant differences between baseline and follow-up, for example, fruit and vegetable consumption increased during the intervention. Other desirable behaviors (eg. decreasing intake of fats or sweets) were also directly rewarded by the program. Moreover, we found that food consumption did differ between normal weight and overweight employees and healthy eating behaviors in the overweight group improved more than in the normal weight group.

These findings are in line with those of a meta-analysis of studies performed in different countries revealed that overweight adults quite frequently have a more unhealthy diet (fat, sweets) than normal weight adults [43]. In contrast, Ortega and colleagues also report that food consumption between overweight and normal weight adolescents did not differ [44].

Although not all of the results reached statistical significance, we demonstrated improvements after a twelve-month follow-up. In that regard, previous evaluation studies demonstrate a high effectiveness and importance of prevention programs on healthy lifestyle changes [19, 33, 45]. However, further investigation is needed on how to achieve change in the broader range of dietary behaviors.

### Subjective health status

In addition, this study’s results do not show any effects on subjective health status neither in overweight nor in normal weight peers which is in line with other studies showing that the effects of health promotion programs increase only over time [46, 47].

This study also suggests that long-term changes in subjective health status require more than physical activity and dietary counseling, including extensive attendance of therapists. It further supports the idea that long-term health prevention in workers may be a more cost- and time effective approach [48].

### Implications for research and practice

This study, aiming at health promotion for employees at a logistics company, did show initial effects. For the future development of worksite health promotion interventions, given the complexity of overweight and obesity, it is recommended to address dimensions other than physical training and combine environmental and individual components in an intervention.

Furthermore, it is recommended for multi-component intervention studies to perform intermediate measurement to gain insight in the possible attrition of effects, if present. In addition, it is suggested to develop and validate more reliable questions in order to conduct differentiated measurements on health behavior at work.

Moreover, long-term results of this study may show the efficacy of a multilevel physical activity program on changes in health behavior and weight gain in (overweight) employees.

Therefore, continuation of this worksite health program is strongly recommended.

### Limitations

This study analyses preliminary results of a worksite intervention targeting physical activity and eating behaviors. This investigation does have some limitations. First, this was not a randomized study. The group was self-selected which may limit the generalizability of results on program effectiveness. This evaluation study is further limited by the nature of the data, which were self-reported. However, with having a twelve months’ time-lag between baseline and follow-up the self-report bias may have been minimized because it can be assumed that participating workers were not able to remember the questions and their responses of the baseline questionnaire. In addition, we collected self-reported height and weight data from the respondents in order to categorize BMI groups. As reported in several studies, self-reports overestimate height and underestimate weight [49, 50]. Consequently, the prevalence of obesity based on self-reported data is underestimated. This may induce a potential bias in our study results. However, distribution of height and weight in this study sample is comparable to that of the population from which the sample was drawn [51], therefore the risk of bias is limited. Due to practical reasons self-reported measurement was the only feasible way of data collection in this large study sample.

## Conclusion

Our findings from the one-year pilot of the intervention program suggest that a worksite health prevention program may improve health behaviors of employees. The program encourages health behavior changes. In conclusion, this study at a logistics company showed that physical activity training combined with nutrition counseling can be implemented successfully during working hours on a long-term basis with initial effects on health behavior changes. Effects on body weight decrease were minimal. Our findings highlight the need for worksite health promotion strategies that provide increased motivation, support and skills to enable employees living their life in a healthy way. Similarly, the results suggest a need to promote time-efficient physical activity options and alternatives. Additional strategies that recognize the perceived barriers to physical activity and healthy eating faced by employees are particularly required.
